# Expressed repetitive elements are broadly applicable reference targets for normalization of reverse transcription-qPCR data in mice

**DOI:** 10.1038/s41598-018-25389-6

**Published:** 2018-05-16

**Authors:** Marjolijn Renard, Suzanne Vanhauwaert, Marine Vanhomwegen, Ali Rihani, Niels Vandamme, Steven Goossens, Geert Berx, Pieter Van Vlierberghe, Jody J. Haigh, Bieke Decaesteker, Jolien Van Laere, Irina Lambertz, Frank Speleman, Jo Vandesompele, Andy Willaert

**Affiliations:** 1 Center for Medical Genetics, Ghent University, Ghent, Belgium; 2 Department of Microbiology, Tumor and Cell Biology, Karolinska Institute, Stockholm, Sweden; 3 Molecular and Cellular Oncology Lab, Inflammation Research Center, VIB, Ghent, Belgium; 4 Department of Biomedical Molecular Biology, Ghent University, Ghent, Belgium; 5 Cancer Research Institute Ghent (CRIG), Ghent, Belgium; 6 Mammalian Functional Genetics Group, Australian Centre for Blood Diseases, Monash University, Melbourne, Australia

## Abstract

Reverse transcription quantitative PCR (RT-qPCR) is the gold standard method for gene expression analysis on mRNA level. To remove experimental variation, expression levels of the gene of interest are typically normalized to the expression level of stably expressed endogenous reference genes. Identifying suitable reference genes and determining the optimal number of reference genes should precede each quantification study. Popular reference genes are not necessarily stably expressed in the examined conditions, possibly leading to inaccurate results. Stably and universally expressed repetitive elements (ERE) have previously been shown to be an excellent alternative for normalization using classic reference genes in human and zebrafish samples. Here, we confirm that in mouse tissues, EREs are broadly applicable reference targets for RT-qPCR normalization, provided that the RNA samples undergo a thorough DNase treatment. We identified *Orr1a0*, *Rltr2aiap*, and *Rltr13a3* as the most stably expressed mouse EREs across six different experimental conditions. Therefore, we propose this set of ERE reference targets as good candidates for normalization of RT-qPCR data in a plethora of conditions. The identification of widely applicable stable mouse RT-qPCR reference targets for normalization has great potential to facilitate future murine gene expression studies and improve the validity of RT-qPCR data.

## Introduction

Reverse transcription quantitative PCR (RT-qPCR) is the gold standard method for quantifying gene transcription. In order to accurately and reproducibly compare mRNA concentrations across different samples, it is essential to normalize the raw data^1^. The preferred normalization strategy is to use expression levels of reference genes that are stably expressed in the conditions used in the study^2^. The Minimum Information for Publication of Quantitative Real-Time PCR Experiments (MIQE) guidelines highlight the importance of properly selecting and validating reference genes for qPCR normalization and suggest testing multiple candidate reference genes for a specific experimental design, followed by using the geometric mean of an experimentally determined optimal number of genes (2 or more) with the most stable expression^1,3^. Failure to validate the applied reference genes is likely to result in misinterpretation of the data^3^.

To date, commercial “endogenous control gene panels” for validation of RT-qPCR mouse reference genes are available. These panels comprise the most commonly used reference genes in mouse research and these genes are considered to be generally stable under a wide variety of circumstances (e.g. across different tissues, mouse strains, experimental conditions, etc.). Various reports, however, have shown that the most popular reference genes are not always the most stable ones^4–6^. Moreover, there is no reference gene that is universally stable and thus suitable for any type of gene expression study^7^.

Recently, our lab pioneered the use of expressed repetitive elements (ERE) as reference targets for RT-qPCR normalization in human and zebrafish samples^8–11^. Multiple copies of these repetitive elements are interspersed throughout the genome and can be located in sequences that are transcribed to RNA. Consequently, a specific ERE will be present in a large number of different transcripts. Differential expression of some of these transcripts in certain circumstances will not substantially influence the total level of ERE expression. Therefore, EREs should be stably expressed throughout different experimental conditions, making them an attractive measure for general mRNA fraction abundance. Indeed, in the zebrafish study, EREs were shown to outperform commonly used reference genes in a variety of experimental set-ups^8^. Likewise, a similar approach using expressed Alu repetitive elements for RT-qPCR normalization in studies on human samples has also proven to be successful^10,12^.

It was hypothesized that the multi-step strategy to identify EREs for RT-qPCR normalization in zebrafish could be applied in other species^8^. In this study, we evaluate this strategy for mouse gene expression studies. We compared the expression stability of mouse ERE reference targets and commonly used reference genes in six different experimental studies. We demonstrate that the murine EREs outperform the frequently used reference genes in a broad range of experimental set-ups.

## Results

### Identification and selection of reference targets in the mouse genome

Candidate ERE reference targets were identified following a slightly adapted workflow previously published for zebrafish^8^. In a first step, 313 EREs present in the mouse genome were identified using the repetitive DNA element database, Repbase (http://www.girinst.org/repbase). Since the number of copies for the different EREs in the mouse genome was unknown in Repbase, initially 16 EREs were selected based on a mean conservation rate of minimum 97% (empirical threshold) compared to the Repbase consensus sequence. The selected EREs include *Bglii_mus*, *Ervb4_1B-LTR_Mm*, *Ervb4_3-I_Mm*, *Ervb7_1-I_Mm*, *Iap1-Mm*, *Iapltr3*, *Iapltr3_i*, *Ltris3*, *Orr1a0*, *Rltr10b2*, *Rltr13a3*, *Rltr13b2*, *Rltr13b3*, *Rltr2aiap_Mm*, *Rltr4i_Mm*, and *Rltr4_Mm*. A blastn search against all mouse RefSeq and non-RefSeq annotated transcripts using these 16 EREs showed that the number of expressed loci ranged between 30 and 400, which was considered an adequate number of hits (more is likely better). Subsequently, primers targeting the most conserved region of these 16 EREs were designed using Primer3 software (http://bioinfo.ut.ee/primer3-0.4.0/).

In parallel, 10 of the most commonly used mouse reference genes (*Actb*, *B2m*, *Gapdh*, *Gusb*, *Hprt1*, *Polr2a*, *Tbp*, *Tuba1a*, *Tubb5*, and *Ywhaz*) and corresponding RT-qPCR primer sequences were selected from literature or RTPrimerDB (http://www.rtprimerdb.org), a public database for primer and probe sequences^13^.

Only primer pairs with an amplification efficiency between 90 and 110% and a coefficient of determination (r^2^) of the standard curve higher than 0.98 were retained, resulting in a final selection of 11 ERE reference targets and 8 reference genes (Table 1).

**Table 1:** Selected EREs and reference gene targets and their respective primer sequences.

Reference target	Forward primer	Reverse primer	Predicted number of targets (a)	Predicted number of targets (b)
**EREs**				
*Ervb4_1B_LTR_Mm*	agcctaataaacgagaccttgat	ccgcgggattcagttattcg	153	151
*Ervb7_1-I_Mm*	aaagtgttgctgaggatgcg	ttccacctaagcagcttcct	117	117
*Iap1_Mm*	tgggaggtatgtctgattgca	tgatccccagtgtgcagaaa	69	69
*Ltris3*	attgctggaacccactatgc	gccccgagtagctgagtaag	62	59
*Orr1a0*	ggttggaatgggtgtgtcac	tggcttacaggttcagaggt	1788	1716
*Rltr2aiap_Mm*	catgtgccaagggtagttctc	gcaagagagagaatggcgaaac	519	506
*Rltr4_Mm*	gtaacgccattttgcaaggc	ccatctgttctttggccctg	0	0
*Rltr4i_Mm*	tcaggacaagggtggtttga	ggcctgcactaccgaaattc	52	52
*Rltr10b2*	ccaatccgggtgtgagaca	ctgactcgccagcaagaac	284	263
*Rltr13a3*	acagactacattccatgccaag	gccaggcaagagttttacac	132	132
*Rltr13b3*	tccggctgtggttttagagt	tgaaaacgcaaagactggca	343	315
**mRNA genes**				
*Actb*	cacacgagccattgttagta	tctcaattgcctttctgact	ND^a^	ND^a^
*B2m*	gggaagccgaacatactgaa	tgcttaactctgcaggcgtat	ND^a^	ND^a^
*Gapdh*	tgtgtccgtcgtggatctga	ttgctgttgaagtcgcaggag	ND^a^	ND^a^
*Hprt1*	cctaagatgagcgcaagttgaa	ccacaggactagaacacctgctaa	ND^a^	ND^a^
*Polr2a*	acttcttgctcaattctttgac	ccaactggtgacagcaa	ND^a^	ND^a^
*Tbp*	ccccacaactcttccattct	gcaggagtgataggggtcat	ND^a^	ND^a^
*Tuba1a*	aaggaggatgctgccaataa	gctgtggaaaaccaagaagc	ND^a^	ND^a^
*Tubb5*	atgccatgttcatcgcttat	ttgttcggtacctacattgg	ND^a^	ND^a^

The predicted number of targets was determined for all EREs using BiSearch (http://bisearch.enzim.hu). The predicted number of targets is given for (a) maximum three mismatches per primer pair and (b) maximum two mismatches per primer pair. Noteworthy, the predicted number of targets is highest in the two most stably expressed EREs as determined by the Rank aggregation analysis.

^a^The predicted number of targets is not determined for the classic reference genes, as primers were designed to target a single cDNA sequence. ND: not determined.

### Reference target expression stability

**Figure 1:**
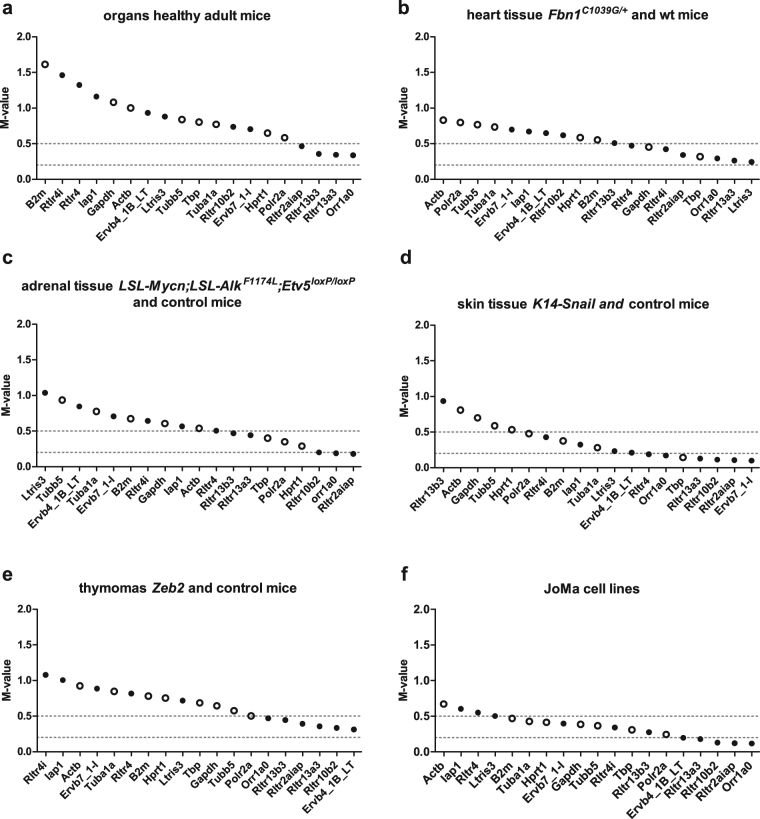
Average expression stability of selected reference genes and ERE reference targets. We determined the mRNA expression level of several classical reference genes and expressed repeat elements in different experiments. The average expression stability for each of the reference targets was calculated using the geNorm algorithm (M-value). Ranking of reference targets depending on their M-values is shown (genes are ordered from left to right according to increasing stability). Cut off values for high (M < 0.5) and very high (M < 0.2) expression stability are indicated by dotted lines. (**a**) Four out of eleven EREs are stably expressed in multiple organs and tissues of wild-type mice (*Orr1a0*, *Rltr13a3*, *Rltr13b3*, and *Rltr2aiap_Mm*). The remaining EREs and all of the classical reference genes have an M-value greater than 0.5 and are therefore less well suited as reference targets in this experiment. (**b**) Seven out of eleven EREs and two out of eight classical reference genes are stably expressed in heart tissue from wild-type and *Fbn1*^*C1039G/*+^ mutant mice. (**c**) In adrenal gland tissue from control and triple transgenic *LSL-MYCN;LSL-ALK*^*F1174L*^*;ETV5*^*loxP/loxP*^*; DBHiCre* mice, the 3 most stable reference targets are EREs (*Rltr2aiap_Mm*, *Orr1a0*, and *Rltr10b2*). Also, 3 classic reference genes (*Hprt1*, *Polr2a* and *Tbp*) show high expression stability, but their M-values are higher than the M-values of the abovementioned EREs. (**d**) All but one ERE (*Rltr13b3*) are stably expressed in the skin from *K14-Snail* transgenic and control mice. Only half of the classic reference genes show ‘high’ to ‘very high’ expression stability. (**e**) None of the classic reference genes have an M-value below 0.5 in the *Zeb2* transgenic mice data set, while six EREs show high expression stability. (**f**) All but three reference targets are stably expressed in the JoMa cell lines. Of those stably expressed reference targets, 5 EREs (*Orr1a0*, *Rltr2aiap_Mm*, *Rltr10b2*, *Rltr13a3*, and *Ervb4_1B_LT_Mm*) are very stably expressed. Commonly used reference genes are represented as open dots (○), ERE reference targets in full black dots (●). wt: wild-type.

The mRNA expression levels of the 11 ERE reference targets and 8 reference genes were assessed in six different experimental conditions. These assays included (1) different organs from healthy adult C57BL/6 mice, (2) heart tissue from wild-type and *Fbn1*^*C1039G/*+^ mutant mice, the latter presenting cardiomyopathy, (3) healthy adrenal tissue and adrenal tumor tissue from control mice and *LSL-MYCN;LSL-ALK*^*F1174L*^*;ETV5*^*loxP/loxP*^; *DBHiCre* quadruple transgenic mice, respectively, (4) skin tissue from *K14-Snail* transgenic mice with precancerous and control skin, (5) p53 null thymomas from *Zeb2*-overexpressing transgenic mice and controls; and (6) four different multipotent neural crest progenitor JoMa1 cell lines (for details and references see materials and methods). The average expression stability value (M-value) of each of the reference targets in the different experiments was calculated using the geNorm algorithm (implemented in the qbase + software). Reference targets with M-values below 0.5 and 0.2 are considered having a ‘high’ and ‘very high’ expression stability, respectively^14^. Overall, the ERE reference targets showed higher expression stabilities (lower M-values) than the commonly used reference genes (Fig. 1).

When comparing the expression stability of the reference targets in multiple organs and tissues of wild-type mice a dispersed M-value distribution was observed. Only EREs (4 out of 11) are highly stably expressed and none of the classical reference genes is stably expressed (Fig. 1a). In heart tissue from wild-type and *Fbn1*^*C1039G/+*^ mutant mice 7 out of 11 EREs and 2 out of 8 classical reference genes are stably expressed (M < 0.5). The three most stably expressed reference targets are EREs (*Ltris3*, *Rltr13a3*, and *Orr1a0*) (Fig. 1b). Similarly, in the *LSL-MYCN;LSL-ALK*^*F1174L*^*;ETV5*^*loxP/loxP*^; *DBHiCre* mice adrenals, the 3 most stable reference targets are also EREs (*Rltr2aiap_Mm*, *Orr1a0*, and *Rltr10b23*) while only 3 classic reference genes (*Hprt1*, *Polr2a* and *Tbp*) show high expression stability (M-value < 0.5) (Fig. 1c). In the skin from *K14-Snail* transgenic mice, a ‘high’ to ‘very high’ expression stability was observed for 10 out of 11 EREs, with 4 EREs being the most stable reference targets (*Ervb7_1-I_Mm*, *Rltr2aiap_Mm*, *Rltr10b2*, and *Rltr13a3*). Half of the classical reference genes also score ‘very high’ (*Tbp*) to ‘high’ (*Tuba1a*, *B2m*, and *Polr2a*) (Fig. 1d). None of the classic reference genes have an M-value below 0.5 in the *Zeb2* transgenic mice data set, while six EREs show high expression stability (Fig. 1e). All but three reference targets are stably expressed in the JoMa1 cell lines (Fig. 1f). Of those stably expressed reference targets, 5 EREs (*Orr1a0*, *Rltr2aiap_Mm*, *Rltr10b2*, *Rltr13a3*, and *Ervb4_1B_LT_Mm*) are very stably expressed (M < 0.2). The relatively low variability in M-values in the latter experiment can be explained by the fact that this sample set is highly homogeneous and that the particular genetic modifications of JoMa1 cell lines does not cause large differences in expression of the investigated reference targets. Nevertheless, the ERE targets showed superior stability compared to the mRNA reference targets.

One of the more frequently used mouse reference genes *Actb*, encoding the beta actin protein, has an M-value greater than 0.5 in all six experiments. *Actb* expression is therefore unstable and, despite its frequent use, not appropriate for normalization in these experimental conditions.

**Figure 2:**
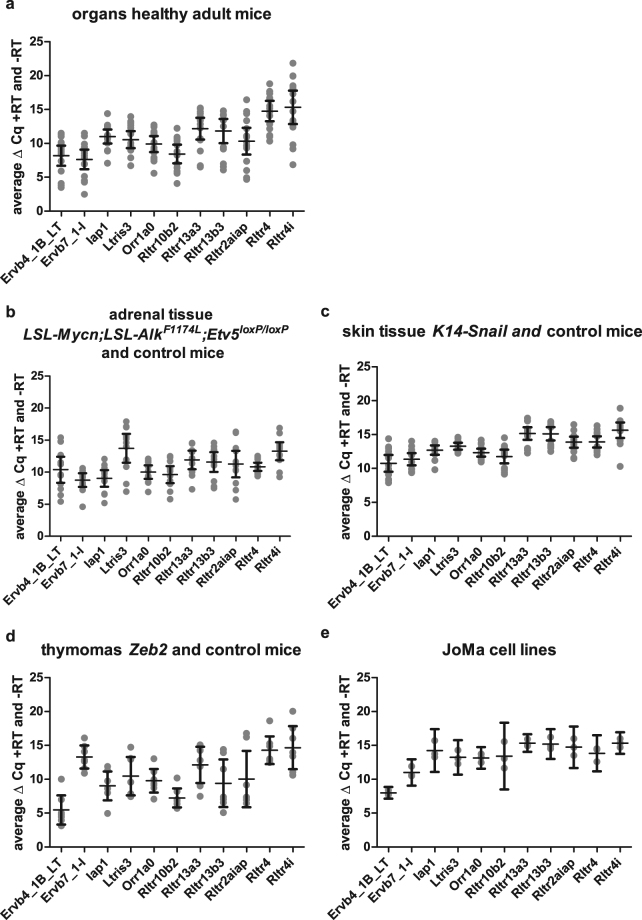
Comparison of the average difference in Cq values between the ERE reference targets of RNA samples treated (+RT) or non-treated (−RT) with reverse transcriptase. Combining on-column and in solution DNase treatment of RNA samples creates an average difference of 11.64 cycles (+/−0.27 (95% confidence interval), range 3 to 21 cycles) when comparing average Cq values of +RT and −RT reactions. This indicates that sufficient amounts of carryover gDNA were removed from the sample in order not to interfere with the RT-qPCR results. Each grey dot represents the difference (number of cycles) in average Cq value of the −RT and +RT reactions of a single sample. Mean and 95% confidence intervals are shown in black.

To exclude genomic DNA interference, we performed qPCR reactions with reverse transcribed or non-treated RNA as input material. We demonstrate that the combination of on-column and in solution DNase treatment of RNA samples results in an average difference of 11.64 cycles (+/−0.27 (95% CI), range 3 to 21 cycles) when comparing average Cq values for the EREs from reverse transcribed versus non-treated RNA input material (Fig. 2).

The geNorm algorithm determined that the optimal number of reference targets to be used for normalization in the different experiments is two, since the V_2/3_ values are lower than 0.15 (see Supplementary Fig. S1). Importantly, in all of the performed experiments the two best (most stable) reference targets with the lowest M-value are EREs.

**Figure 3:**
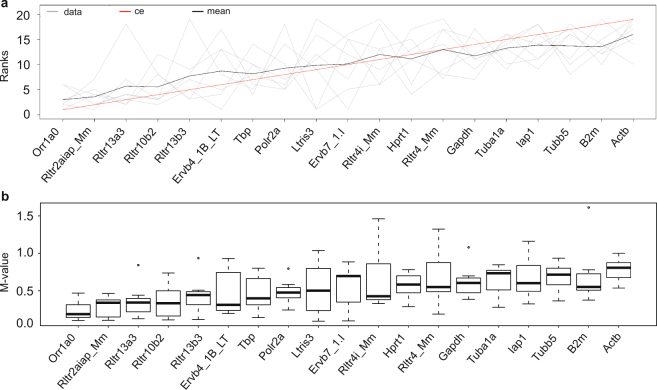
Order of reference target stability. (**a**) Rank aggregation analysis indicates that six out of eleven EREs are more stably expressed than the first ranked reference genes (*Tbp* and *Polr2a*). *Orr1a0, Rltr2aiap*, and *Rltr13a3* (on the left) are the most stable reference targets. Stability measurements are depicted in grey, mean rank position in black and the model computed by the Monte Carlo algorithm in red. (**b**) Boxplot representation of the variation in M-value. The reference targets are ordered according to expression stability (most stably expressed targets on the left). Boxes represent the first and third quartile. Median values are indicated with a line, and outliers with open circles.

In order to identify the most stable reference targets across all experiments performed, a rank aggregation analysis was carried out. This analysis showed that the expression stability of most of the EREs is higher compared to the commonly used reference genes (Fig. 3a,b). The most stable EREs are *Orr1a0*, *Rltr2aiap*, and *Rltr13a3*. Moreover, these 3 EREs had an M-value below 0.5 in each of the 6 experiments. *Orr1a0* is in the top 3 most stably expressed reference targets in 4 out of 6 experiments. *Rltr13a3* and *Rltr2aiap* are in the top 3 most stably expressed reference targets in half of the experiments (3/6). Hence, these EREs are excellent candidates to be evaluated as reference targets in a variety of mouse experiments.

## Discussion

The cornerstone of reliable RT-qPCR results is the use of an adequate number of (typically three) validated and stably expressed reference genes for data normalization. It has been reported that there is no single gene that is stable in all experimental conditions^7^. Differential expression of reference genes is inherent to different tissues and organs, and following perturbations such as tamoxifen-induced gene knock-out, transgene expression, environmental exposures, therapeutic treatments, etc. Hence, it is paramount to determine the validity of the reference genes for normalization of RT-qPCR data in every single experiment. Previously, we showed that the expression of human Alu repeats and zebrafish expressed repeat elements (EREs) are highly stable across different experimental treatments, tissues and developmental stages, hereby outperforming the commonly used reference genes^8^. The highly stable expression of EREs in different experimental conditions is due to the fact that multiple copies of the EREs are dispersed over the genome and that their overall expression is not substantially influenced by differential expression of some of the genes in which the EREs are located. Vanhauwaert *et al*. showed that, in zebrafish, a limited set of three ERE reference targets was suitable for RT-qPCR normalization throughout the wide range of experimental conditions tested^8^. Analogously, in this study, we aimed to identify a set of murine reference targets that are stably expressed in a wide range of experimental conditions and that can be applied for proper RT-qPCR normalization.

We compared the expression stability of 11 ERE sequences and 8 commonly used reference genes in a diverse set of experimental set-ups, including different organs of healthy adult mice, heart tissue of wild-type and *Fbn1* mutant mice presenting cardiomyopathy, normal versus tumor tissue of three different transgenic mouse models (*K14-Snail*, *LSL-MYCN;LSL-ALK*^*F1174L*^*;ETV5*^*loxP/loxP*^*; DBHiCre*, and *Zeb2*), and in multipotent neural crest progenitor JoMa1 cell lines. These experimental set-ups cover different aspects of mouse research in different organs and tissues, as well as a mouse neural crest cell line. Overall, EREs scored better as reference targets for RT-qPCR normalization than the commonly used reference genes. In all of the 6 experimental settings, we could always identify EREs with a high to very high expression stability, represented by a low M-value. In the experiment using different organs from adult wild-type mice none of the commonly used reference genes have an M-value below 0.5 and are therefore not very well suitable for normalization of the data. This setup is characterized by the most dramatic changes in expression profiles and hence represents the most heterogeneous experimental setup studied here. But even in the studies with fairly homogeneous samples, the most stable commonly used reference genes are always inferior to several EREs that are being more stably expressed. Thus, murine EREs reference targets are more suitable as proper reference genes for RT-qPCR normalization across a wide range of murine gene expression studies.

In our study, we have not tested all commonly used reference targets, for example we did not assess expression stability of 18S or other ribosomal RNA targets in our study conditions. Therefore, it is difficult to conclude at the present moment whether such rRNA reference targets are better or worse than the proposed ERE-based normalization strategy. Future research is needed to formulate clear opinions on the applicability of commonly used reference targets that were not tested in the current study for RT-qPCR normalization.

An important concern that needs to be taken into account when using EREs as RT-qPCR reference targets is that it is virtually impossible to design assays that avoid genomic DNA co-amplification. Genomic DNA contamination can be tested by performing a qPCR control reaction using RNA input material that has not been converted into cDNA using reverse transcriptase (−RT control). If the Cq value for the −RT control is considerably higher than for the reverse transcriptase-treated (+RT) sample, this indicates that gDNA does not substantially contribute to the signal. We show that the combination of on-column and in solution DNase treatment of RNA samples is sufficient to exclude gDNA contamination. Although there is some trace amplification of contaminating genomic DNA in a minority of samples, the difference in Cq values is sufficiently high for this not to be a problem when interpreting the RT-qPCR results, since the residual DNA signal contributes on average less than 0.031% (1/2^11.64^) (range 0.000048–12.5%). Therefore, we recommend to conduct a thorough DNase treatment of the RNA samples, preferably applying a combination of both on-column and in solution DNase treatment, when using ERE reference targets for RT-qPCR normalization. These treatments are straightforward and require little extra hands-on time.

Rank aggregation analysis identified six EREs as the most stably expressed reference targets across the six conditions tested in this study. More specifically, *Orr1a0*, *Rltr2aiap*, and *Rltr13a3* are the top 3 most stable reference targets.

Based on these results we recommend including at least these 3 ERE reference targets when validating reference targets for qPCR normalization. The identification of widely applicable stable mouse reference targets for RT-qPCR normalization has great potential to facilitate future gene expression studies and improve the validity of the ensuing results.

## Methods

### Mice

Mice were kept in accordance with the institutional guidelines regarding the care, housing and use of laboratory animals. All procedures were approved by the Ethics Committee for the care and use of laboratory animals of the Ghent University (permit numbers: ECD10/20; ECD07/04; EC2011-013; EC2007-022; EC2014-072).

Several different mouse models were used in this study: (1) wild-type C57Bl/6 female mice; (2) Marfan mice (*Fbn1*^*C1039G/*+^)^15^ presenting cardiovascular manifestations such as cardiomyopathy and wild-type controls; (3) *LSL-MYCN;LSL-ALK*^*F1174L*^*;ETV5*^*loxP/loxP*^*; DBHiCre* quadruple transgenic mice presenting neuroblastoma and their controls (mice lacking the DBHiCre transgene); (4) mouse model with skin-specific overexpression of *Snail* (*K14-Snail* transgenic mouse) characterized by distinct subtypes of skin cancer^16^ and wild-type controls; and (5) heterozygous and homozygous *Zeb2*-overexpressing transgene mice crossed into a tumor-prone p53 floxed background, as a model for early T-cell precursor leukaemia (Tie2cre; p53^fl/fl^; R26-Zeb2^tg/+ or tg/tg^ (P53/R26-Zeb2^tg/+ or tg/tg^)) and their controls (Tie2cre; p53^fl/fl^, (P53/R26^+/+^))^17^. In addition, one mouse cell line was included. JoMa1 is a multipotent neural crest progenitor cell line. A tamoxifen-activated c-Myc transgene (c-MycER(T)) keeps the cell line in an undifferentiated state.

### Dissection of organs from adult wild-type mice

Four 3 months old male wild-type C57Bl/6 mice were sacrificed by means of CO_2_ inhalation. Organs were flushed with 1x PBS. Lungs, liver, kidney, testis, and colon were dissected and snap frozen in liquid nitrogen and stored at −80 °C until RNA-extraction.

### Dissection of hearts from wild-type and Fbn1^C1039G/+^ mutant mice

In the context of a previous study, 7 *Fbn1*^*C1039G/*+^ and 9 wild-type mice were euthanized at the age of 14 months by means of CO_2_ inhalation^18^. Hearts were flushed with 1x phosphate buffered saline (PBS), dissected and snap frozen in liquid nitrogen. Samples were stored at −80 °C until RNA-extraction.

### Dissection of adrenal gland tissue from LSL-MYCN;LSL-ALK^F1174L^;ETV5^loxP/loxP^; DBHiCre quadruple transgenic mice

All transgenic mouse models have been previously described^19–22^. Briefly, in the conditional transgenic LSL-*MYCN* and LSL-*ALK*^*F1174L*^ mice the human *MYCN* and *ALK*^*F1174L*^ genes are cloned downstream of a chicken actin gene (CAG) promotor followed by loxP-flanked strong transcriptional termination site (LSL), which prevents transgene expression in the absence of Cre recombinase^19,20^. In the *Etv5*^*loxP/loxP*^ mice, loxP sites flank the N-terminal portion of the DNA binding domain. Cre-mediated recombination of the *ETV5* floxed exons leads to loss of ETV5 protein expression^21^. The DBHiCre mouse strain carries Cre recombinase under the *Dbh* promoter, confirming expression in the sympathoadrenal neuronal lineage of the sympathetic nervous system^22^. Targeted expression of *MYCN* and *ALK*^*F1174L*^ in the sympatoadrenal lineage leads to development of neuroblastoma tumors, as previously described^19,20^. Four P12-P14 *LSL-MYCN;LSL-ALK*^*F1174L*^*;ETV5*^*loxP/loxP*^*; DBHiCre* mice were euthanized by cervical translocation, followed by isolation of adrenal tumors. Four and six control adrenals were collected from mice lacking the DBHiCre transgene, *LSL-MYCN;LSL-ALK*^+/+^*;ETV5*^*loxP/*+^ and *LSL-MYCN;LSL-ALK*^*F1174L/*+^*;ETV5*^*loxP/*+^, respectively. Tissue samples were rinsed in PBS, snap frozen in liquid nitrogen and stored at −80 °C until further processing.

### Dissection of skin from K14-Snail transgenic mice

The *K14-Snail* transgenic mice have been previously described by De Craene *et al*.^16^. Seven *K14-Snail* pups and seven wild-type pups were decapitated at postnatal day 3, and their limbs and tail were removed. The body was subsequently disinfected in a povidone-iodine solution (iso-Betadine®) and washed twice in 70% ethanol and three times in PBS. The skin was carefully dissected followed by removal of fat tissue using a scalpel. Skin pieces were incubated in RNAlater at 4 °C overnight. Subsequently, skin pieces were put in a petridish in 1 ml TRISure on ice and cut into small pieces. Skin pieces were homogenized using a mechanical mixer followed by passing the sample five times through a 21-gauge needle. To reduce the carryover of RNases during further processing, the homogenized sample is centrifuged for 15 minutes, 20 000 × g, 4 °C to eliminate debris. The supernatant is transferred to a new RNAse-free eppendorf and incubated for 5 minutes at room temperature. After phase separation by adding 200 µl chloroform and vigorous shaking, 250 µl from the aqueous upper phase was added to 500 µl cold RNase-free ethanol. Samples were snap-frozen in liquid nitrogen and stored at −80 °C.

### Dissection of thymomas from Zeb2-overexpressing transgenic mice

The *Zeb2*-overexpressing transgenic mice have been previously described by Goossens *et al*.^17^. Thymomas were harvested from five P53/R26-Zeb2^tg/tg^, five P53/R26-Zeb2^tg/+^, six P53/R26^+/+^ mice and incubated in sterile cold PBS. Tissues were processed for single cell suspension on a 40 µm filter mesh and washed once with PBS. Cells were collected via centrifugation (5 min, 1 300 rpm, 4 °C) and cell pellets were snap frozen in liquid nitrogen and stored at −80 °C until RNA-extraction.

### Selection JoMa1 cell lines

Three different JoMa1 cell lines^23,24^ were used, JoMa1 (n = 3), JoMa1-MYCN (n = 2), and JoMa1-GFP (n = 1). JoMa1-MYCN cells express the oncogene *MYCN*, this enables the cells to grow independently of c-MycER(T) activity *in vitro* and to form neuroblastoma-like tumors *in vivo*. JoMa1-GFP cells express GFP, but do not have tumor forming potential *in vivo*.

### RT-qPCR

RNA isolation from frozen tissue or cell pellets was performed using the RNeasy Mini or RNeasy Plus Mini (*Zeb2* and *K14-Snail* transgenic mice) kit (Qiagen) in combination with on-column DNase I treatment using the RNase-Free DNase kit (Qiagen) according to the manufacturer’s guidelines. Following RNA isolation, samples were subjected to an additional DNase treatment using the Heat & Run gDNA removal kit (ArcticZymes) per manufacturer’s recommendations. RNA concentrations were measured with the NanoDrop spectrophotometer (Thermo Scientific) or DropSense96 (Trinean). RNA integrity was assessed using an Experion automated electrophoresis system (Bio-Rad) or Agilent 2100 Bioanalyser (Agilent). Only RNA samples with an RQI- or RIN-score equal or above 7 and which showed no signs of degradation on the electropherogram pattern were used for further analysis (n = 3-4/genotype or organ or treatment group). cDNA was synthesized using the iScript kit (Bio-Rad) according to the manufacturer’s guidelines. RNA that was not subjected to reverse transcriptase PCR was used as control to assess gDNA contamination. Subsequently, RT-qPCR was carried out as described previously^8^. Primer sequences (Table 1) were designed using Primer3 software (http://bioinfo.ut.ee/primer3-0.4.0/) and amplification efficiencies were tested using a standard dilution series (6 points, dilution factor ¼, maximum input 10 µg) made from RT-qPCR mouse reference total RNA.

### Statistics and data analysis

Statistics and data analysis was performed as previously described^8^. In short, the geNorm module in qbase^+^ version 2.6.1 (Biogazelle, http://www.qbaseplus.com) was used to calculate expression stability values for all reference targets (M-value) and to determine the optimal number of reference targets for every experiment (V-value). Graphs were created using GraphPad Prism version 5.04.

The Rankaggreg package in R (version 3.2.3) was used to perform rank aggregation analysis.

### Data availability

The datasets generated during and/or analysed during the current study are available from the corresponding author on reasonable request.

## Electronic supplementary material

Supplementary information
