# Supplementary material for: Expressed repetitive elements are broadly applicable reference targets for normalization of reverse transcription-qPCR data in mice
Source: Sci Rep. 2018 May 16;8:7642. doi: 10.1038/s41598-018-25389-6 (PMC5955877; doi:10.1038/s41598-018-25389-6)
Supplement: Supplementary file 1 — Supplementary information [file 41598_2018_25389_MOESM1_ESM.pdf]

Supplementary information

Expressed repetitive elements are broadly applicable reference targets for normalization of reverse transcription qPCR data in mice

Marjolijn Renard[1], Suzanne Vanhauwaert[1], Marine Vanhomwegen[1], Ali Rihani[2], Niels Vandamme[3, 4, 5], Steven Goossens[1, 3, 4, 5], Geert Berx[3, 4, 5], Pieter Van Vlierberghe[1, 5], Jody J. Haigh[6], Bieke Decaesteker[1], Jolien Van Laere[1], Irina Lambertz[1], Frank Speleman[1, 5], Jo Vandesompele[1, 5], Andy Willaert[1,*]

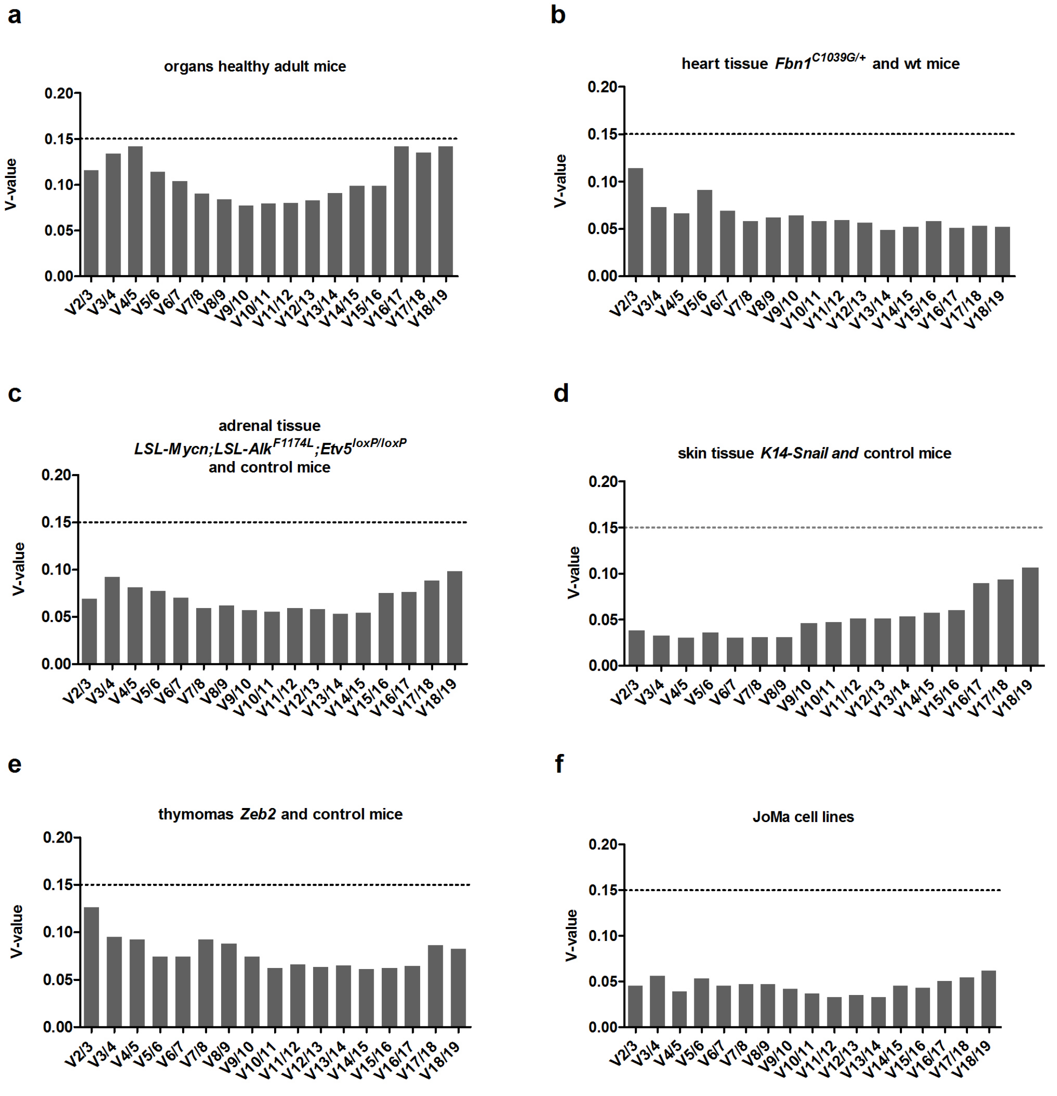

Supplementary figure S1: Determination of the optimal number of reference targets. Pairwise variation V_n/V_{n+1} values were calculated for the six different experimental conditions using geNorm. The optimal number of reference targets (n) is reached when the inclusion of the next target (n+1) reduces the V-value below 0.15 (cut off value indicated by a dotted line). In each of the six different experiments inclusion of the two most stable reference targets (lowest M-value) is sufficient for normalization of the data, since the $V_{2/3}$ values are lower than 0.15. wt: wild-type.
